# A Review of Cu–Ni–Sn Alloys: Processing, Microstructure, Properties, and Developing Trends

**DOI:** 10.3390/ma16010444

**Published:** 2023-01-03

**Authors:** Lang Guo, Pengcheng Zuo, Zequn Zhang, Qianwen Zhang, Mengya Zhao, Xinyu Hou, Junsheng Wu, Bowei Zhang

**Affiliations:** Institute for Advanced Materials and Technology, University of Science and Techonlogy Beijing, Beijing 100083, China

**Keywords:** Cu–Ni–Sn, alloy, processing, microstructure, property

## Abstract

Cu–Ni–Sn alloys have been widely used in the aerospace industry, the electronics industry, and other fields due to their excellent electrical and thermal conductivity, high strength, corrosion and wear resistance, etc., which make Cu–15Ni–8Sn alloys the perfect alternative to Cu–Be alloys. This paper begins with how Cu–Ni–Sn alloys are prepared. Then, the microstructural features, especially the precipitation order of each phase, are described. In addition, the influence of alloying elements, such as Si, Ti, and Nb, on its microstructure and properties is discussed. Finally, the effects of plastic deformation and heat treatment on Cu–Ni–Sn alloys are discussed. This review is able to provide insight into the development of novel Cu–Ni–Sn alloys with a high performance.

## 1. Introduction

Copper and copper alloys are some of the most important materials in new-generation information technology, new energy vehicles, rail transit equipment, aerospace and ocean engineering, and other fields [1]. Among the copper alloys, the Cu–Be alloy used to play an indispensable role in the economic industry and was called the “king of nonferrous elastic materials” [2]. However, the production of Cu–Be alloys generates toxic dust, and their stress relaxation performance and conductive stability tend to dramatically deteriorate at high temperatures. A costly and complex preparation technology is required to address these issues, which limits their applications [3]. With the rapid movement of the electronics industry toward miniaturization, integration, and high reliability, it is of great significance to prepare high-strength and elastic copper alloys that are safe, non-toxic, simple to prepare, cost-effective, and provide a good service performance. A series of beryllium-free and high-performance copper-based elastic alloys have been successfully developed, such as Cu–Ti [4], Cu–Ni–Sn [5], Cu–Ni–Mn [6], Cu–Ni–Al [7], and Cu–Fe–P [8].

**Figure 1:**
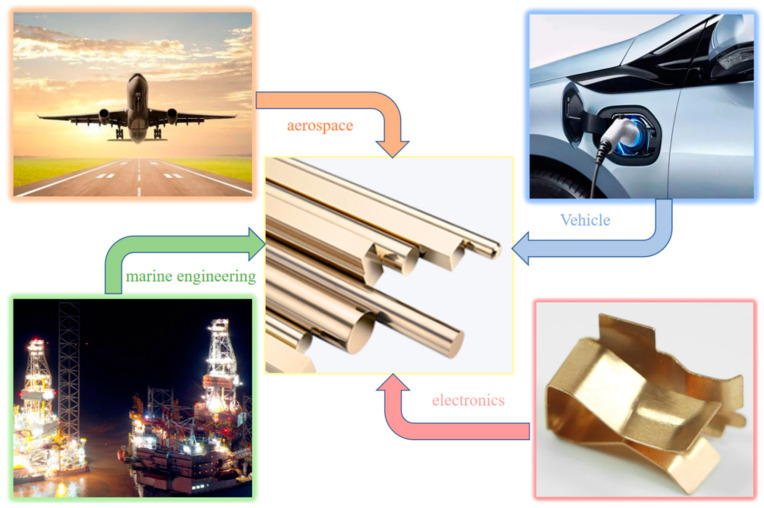
Application of Cu–Ni–Sn alloys.

Since their successful development by Bell Telephone Laboratories in the 1970s, Cu–Ni–Sn alloys have received widespread attention [9]. The main Cu–Ni–Sn alloys are Cu–9Ni–2Sn (UNS C72500), Cu–4Ni–4Sn (UNS C72600), Cu–9Ni–6Sn (UNS C72700), Cu–10Ni–8Sn (UNS C72800), and Cu–15Ni–8Sn (UNS C72900). Among them, Cu–15Ni–8Sn has the best wear resistance and the highest mechanical strength and hardness [10]. The mechanical properties and wear resistance of Cu–Ni–Sn alloys are similar to those of Cu–Be alloys, while the thermomechanical workability and corrosion resistance of Cu–Ni–Sn alloys are better than those of Cu–Be alloys [11]. As an excellent spring material, Cu–15Ni–8Sn alloys are used as friction materials for manufacturing high-performance aerospace bearings, tooth wheel drill bits, and heavy-duty mobile industrial equipment, and have great prospects for application in bearing materials in, for example, the automotive, aircraft, drilling equipment, and machine tool industries [5]. Figure 1 shows a partial application of Cu-Ni-Sn alloy.

After reviewing the existing literature on Cu–Ni–Sn alloys, in this paper, we first introduce the development of Cu–Ni–Sn alloys in the past decade or so and briefly describe the preparation process of the alloys, including continuous casting, powder metallurgy, and the plasma spraying and rapid solidification methods. Specifically, we summarize the application of a promising rapid solidification method, laser powder bed fusion (LPBF), in the preparation of Cu–Ni–Sn alloys. The strengthening mechanism of the high-strength copper alloy, Cu–15Ni–8Sn, is discussed in detail, followed by a discussion of the effects of adding different alloying elements on the structure of the alloy. Finally, we illustrate the influence of plastic deformation and heat treatment on the microstructure and properties of Cu–Ni–Sn alloys.

## 2. Microstructure of Cu–Ni–Sn Alloys

**Figure 2:**
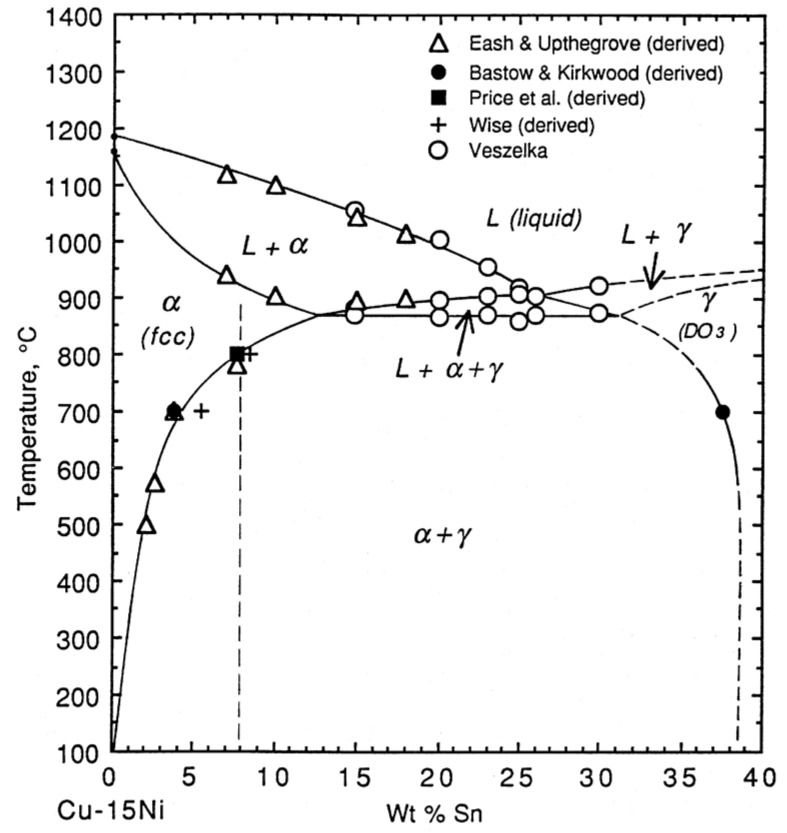
Cu–Ni–Sn ternary phase diagram (Ni fixed at 15 wt%) [12].

Cu–15Ni–8Sn is a typical precipitation hardening alloy. The strengthening mechanism is the spinodal decomposition of the supersaturated solid solution. When Sn is added, at least six different precipitation products can be formed in the aging process: the γ (DO_3_) phase, which precipitates at the grain boundaries or in the grains, the ordered DO_22_ phase ((Cu_x_Ni_1-x_)_3_Sn), the L1_2_ phase, the spinodal-decomposition-induced amplitude modulation structure, and the orthogonal β phase. Among them, the most studied phase is the metastable (Cu_x_Ni_1-x_)_3_Sn phase with a DO_22_ lattice structure during spinodal decomposition. Figure 2 presents the ternary phase diagram of Cu–Ni–Sn. Since the content of Ni is fixed at 15 wt%, the diagram can actually be regarded as a binary phase diagram. The dotted line in the figure indicates that Sn is 8 wt%. At this composition, the alloy begins to precipitate the α phase (FCC) from the liquid phase when the temperature drops below 1150 °C. As the temperature decreases further, more α phase is formed. Sn atoms precipitate continuously in the form of a γ solid solution in the α phase, leading to precipitation strengthening. The γ phase is an important factor affecting the properties of the alloy. Different solution temperatures, times, and cooling rates directly influence the size, shape, and distribution of the γ phase precipitates.

**Figure 3:**
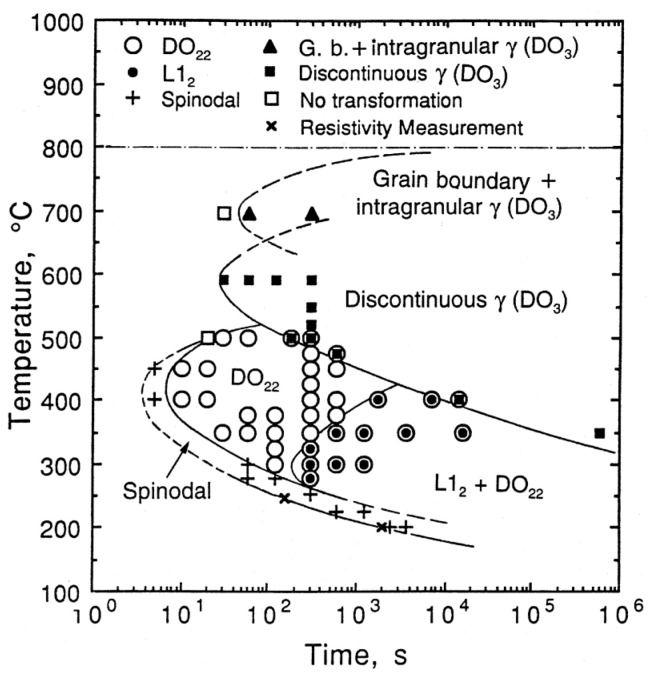
TTT diagram of the alloy Cu–15Ni–8Sn [12].

Zhao et al. [12] established a detailed TTT diagram (see Figure 3) for the isothermal decomposition of the alloy Cu–15Ni–8Sn (wt%) based on a large number of TEM characteristic and resistivity measurements. According to the diagram, amplitude modulation decomposition does not occur above 500 °C and the precipitation from the matrix during tempering at a higher temperature (600 °C) is the discontinuous γ (DO_3_) phase. With an extension of annealing time or a decrease in temperature, the ordered DO_22_ phase with a typical Al_3_Ti intermetallic crystalline structure begins to precipitate at 500~350 °C, followed by the further precipitation of the ordered L1_2_ phase. Finally, when the temperature drops below 300 °C, spinodal decomposition occurs along with the simultaneous appearance of a mixed-phase structure of DO_22_ and L1_2_.

**Figure 4:**
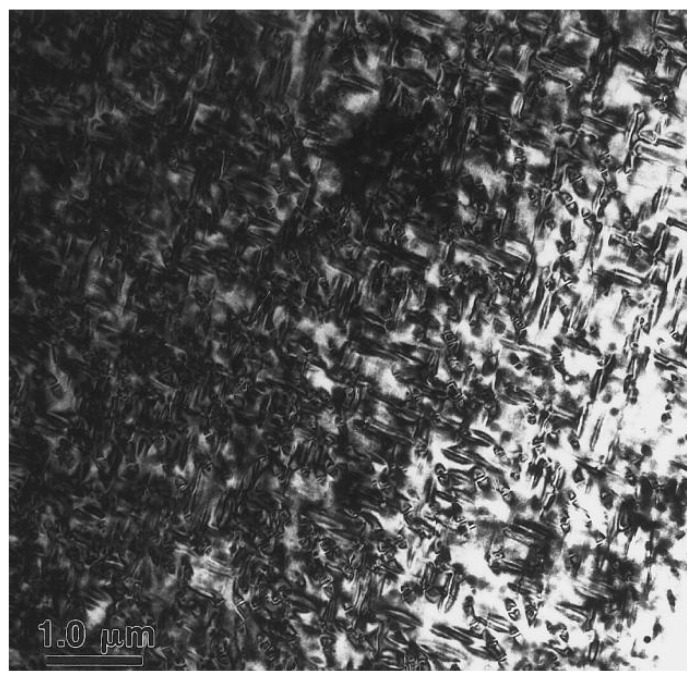
Bright-field (BF) TEM image of a quenched Cu–15Ni–8Sn sample [12].

Figure 4 is a bright-field (BF) TEM image of a quenched sample of a Cu–15Ni–8Sn alloy. After the sample is aged at 350 °C for 60 min, a typical spinodal decomposition nanostructure is formed. The Sn-rich region is filled with the skeleton of the amplitude modulation structure, which is interspersed with the surrounding Sn-poor region to form a braided nanostructure. The aging temperature determines the absence of discontinuous precipitation of γ. In [13], it is suggested that discontinuous precipitation will occur after the spinodal decomposition in a prolonged aging process, leading to a decrease in yield stress. In addition, a study carried out by Luo [14] suggested that the discontinuous precipitation of the γ (DO_3_) phase can also be achieved by the decomposition of DO_22_ precipitated after spinodal decomposition.

## 3. Preparation Process

Continuous casting, powder metallurgy, plasma spraying, rapid solidification, and vacuum melting are some of the common methods of preparing Cu–Ni–Sn alloys.

### 3.1. Continuous Casting

Continuous casting, an economical and efficient technology, is now widely used to manufacture copper-based materials. For copper alloys with a high Sn content, it is common for the Sn to segregate during the continuous casting process and it is difficult to completely eliminate this Sn in the subsequent heat treatment, leading to the degradation of the mechanical properties and corrosion resistance of the alloys. There are two types of continuous casting: horizontal casting and vertical casting.

In the case of horizontal casting, electromagnetic stirring technology is added and the molten metal with current-carrying conductive properties will form a large electromagnetic force with the action of an external electromagnetic field, which results in the linear or rotational movement of the molten metal. The essence of electromagnetic stirring technology is using electromagnetic force to compel the molten metal to move in a specific direction. At the same time, the temperature and concentration fields of the molten metal are made uniform to reduce the nucleation work and critical nucleation radius and increase the number of equiaxed crystals during the solidification process, which can help achieve grain refinement and improve the quality of the cast billet [15]. Horizontal casting solves the problem of uneven solidification of liquid metal caused by gravity, refines the grain of billet [16], and improves the quality of continuous casting billet. Moreover, the bridges are formatted by the unbalanced growth of dendrites, which prevents the upper metal liquid from shrinking downward, thus forming a central shrinkage. However, the dendritic growth conditions can be improved effectively [17].

The advantage of vertical casting is that it uses the additional factor of gravity and the technology of hot top so that the copper liquid can be injected uniformly at uniform speed and temperature, so as to produce qualified billets. However, at present, electromagnetic stirring technology is mostly applied to tube billets and the application of slab, square, and round billet continuous casting is not mature [18]. Numerical simulation can also be used to provide data support for this technology in the future.

### 3.2. Powder Metallurgy

In powder metallurgy, metal powder or a mixture of metal powder and non-metallic powder is used as the raw material to manufacture metal materials, composites, and various products through forming and sintering. The basic process of preparing alloys by the powder metallurgy method is as follows: First, some pre-alloyed powder is prepared by atomization. Then, the alloy is prepared by conventional powder metallurgy processes, such as pressing, forming, sintering. An alloy prepared by powder metallurgy is more homogeneous, and the segregation of Sn in Cu–Ni–Sn alloys can partially be suppressed. To prevent the addition of Ni from affecting the solid solubility of Sn in Cu, and to maximize the solid solubility of Sn in Cu, there are generally two ways of obtaining raw material powder: one is to add Cu powder or Ni powder into the partial pre-alloying powder composed of the binary alloy powder tin bronze, and the other is to obtain Cu–Ni–Sn ternary pre-alloying powder by rapid solidification atomization. In both methods, the alloyed Sn is added to the raw material powder so that Sn segregation is restrained to a certain extent. Therefore, even for alloys with a high Sn content, such as C72900 and C72800, Cu–Ni–Sn alloys with uniform composition can be successfully prepared by this method [19]. Then, the raw material powder above undergoes different forming and sintering processes. For example, ternary pre-alloyed powders are capable of being directly rolled into strips through a continuous production line. Conventional methods can also be used for press and sinter forming. The operation involves mixing the lubricant into the powders and pressing the mixture into shape under water pressure. Then, the intermediate product is kept at a certain temperature for a period of time so the lubricant is fully volatilized. Finally, sintering is carried out to produce the compact sample. In the sintering process, the step-by-step sintering method can be used to prevent shape changes in the pressed billet due to uniform heating. In the so-called step-by-step sintering method, the blanks are kept in a low-temperature range for a specific time period and then heated up to the high-temperature zone for sintering.

Experiments have proved that compared to normal isothermal sintering, when the step-by-step sintering method is used, sintered samples with lower porosity, higher relative density, and more homogeneous composition can be obtained. Wu [20] prepared a Cu/invar composite using powder metallurgy and investigated a series of preparation parameters, such as optimal forming pressure and sintering temperature, achieving further enhancements in electrical and thermal conductivity.

The advantages of preparing Cu–Ni–Sn alloys with the powder metallurgy method are as follows [21]: (1) To a certain extent, the problem of Sn segregation in the preparation of Cu–Ni–Sn alloys with a high Sn content using the traditional smelting method is overcome. (2) The alloy composition can be controlled more precisely; a more homogeneous composition and finer grain size can be obtained for the subsequent age-hardening treatment. However, at present, the process still has shortcomings due to the high production cost. Furthermore, this method is only used to produce some products with simple shapes, such as strips and wire rods.

### 3.3. Plasma Spraying

Plasma spraying strengthens the surface of the material to enhance its wear and corrosion resistance. The method is also effective for preparing copper alloy coatings. A semi-solid spray droplet is deposited on the formed substrate, casting a homogeneous organization. Xiao [22] used thermal-spraying technology to prepare a Cu–15Ni–8Sn alloy coating, and the optimal arc current was experimentally derived. The results of the studies show that under the best arc current conditions, the number of the nonmelted or semi-molten particles is reduced and the wear resistance of the coating is greatly improved after aging. Low-segregation, fine-grain, high-density billets close to the final forming size from liquid metal rapid solidification can be directly prepared by this method, which can effectively inhibit the diffusion and aggregation of alloy elements and solve the problem of Sn reverse segregation. However, the manufacturing cost is high, the size of the billet should not be too large, and the microstructure of the bottom and middle positions of the alloy’s deposited billet varies greatly.

### 3.4. Rapid Solidification Methods

**Figure 5:**
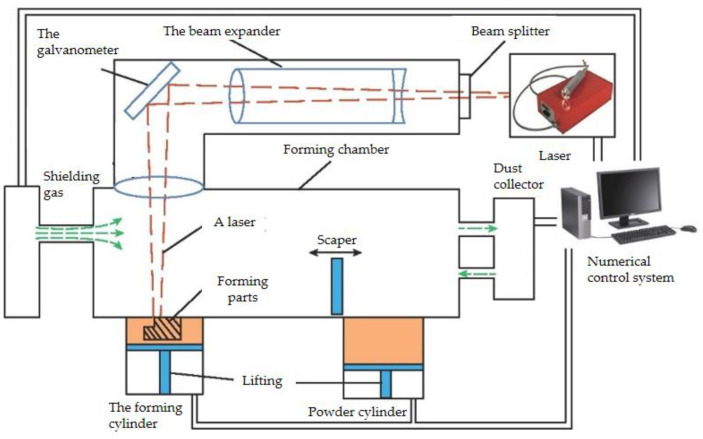
LPBF principle [27].

Rapid solidification refers to a solidification process with a solidification rate much faster than that of conventional casting (generally faster than 10 mm/s). It is a non-equilibrium solidification process that usually produces a metastable phase, resulting in a material with excellent strength, hardness, and corrosion resistance properties. Laser powder bed fusion (LPBF; Figure 5), which is one of the main technical approaches in the additive manufacturing of metallic materials, represents a rapid solidification method. In a protective atmosphere, the metal powder is completely melted by a high-energy laser beam along the laser path, followed by rapid solidification of the molten metal. By repeating this step, the layers are stacked, resulting in a high-density, high-performance metal three-dimensional component. Compared with traditional processing methods, LPBF has many advantages [23]: during the LPBF forming process, the laser scanning rate and the solidification cooling rate of the powder are fast, resulting in a fine and uniform grain structure of the formed sample, and the segregation of elements can be effectively suppressed, which improves the comprehensive performance of the formed components. LPBF technology is a layer-by-layer stacking technique, making it possible to build prototypes with complex structures that are difficult to process by conventional methods. Compared with the traditional preparation method, the process is relatively simple. The material utilization rate is high, and the unformed powder can be recycled. In Cu–Ni–Sn alloys of C72500 and C72900, macrosegregation is eliminated by rapid solidification but microsegregation still exists in thin strips. Rapid solidification significantly reduces the degree and spacing of segregation [24]. In addition, due to the fast solidification rate, the grain can be significantly refined, and it has been claimed that Cu–Ni–Sn alloys can be prepared without segregation through the design of preparation parameters [25]. Cu–15Ni–8Sn alloys prepared by LPBF have good strength and toughness, an RM of 593.3 MPa, and an elongation, a, of 19.8%. The elastic modulus, E, of the LPBF alloy is 144 GPa, which is 7% higher than that of the as-cast alloy. Complex-shaped parts can be manufactured by LPBF technology, which is another advantage [26].

Nevertheless, LPBF technology is still in the research stage due to its high cost, and has many problems: During the LPBF-forming process, large residual internal stress is generated in the formed components by the rapid cooling and solidification process of metal materials, which can lead to cracks in the material. Depending on the construction direction and scanning strategy of LPBF technology, directional growth of grains can be caused by the large temperature gradient and complex heat transfer caused by the laser, and as a result of this, the microstructure and properties of the alloy tend to be anisotropic. In addition, the quality of parts obtained by LPBF depends on the selection of process parameters, such as laser power, scanning speed, scanning spacing, and layer thickness, which are influenced by material properties, such as powder fluidity, particle size, shape, and distribution, as well as the type and spot size of laser. Finally, inappropriate process parameters can cause defects such as pellets, pores, cracks, and low density, significantly reducing the comprehensive performance of LPBF parts.

### 3.5. Vacuum Melting Process

In vacuum melting, alloys are melted under the protection of vacuum or other gases (hydrogen, argon, etc.) which can strictly control the composition of the alloy and ensure that the material has a high degree of purity. During the melting process, the segregation of Sn is not reduced by the vacuum melting process itself [28]. Therefore, various trace alloying elements (such as Fe and Si) are added during the melting process, or the ingot is homogenized after melting to reduce the segregation of Sn and improve the alloy properties.

Guo et al. prepared a Cu–15Ni–8Sn alloy by the vacuum melting method and investigated the effect of adding Fe on the properties [29]. The results showed that the addition of Fe to the Cu–15Ni–8Sn alloy could improve the alloy’s anti-aging resistance, hardness, and tensile properties. Furthermore, when the Fe content was 0.1%, the maximum ultimate tensile strength of the alloy was 123 MPa higher than that of the Fe-free alloy.

The advantages and disadvantages of the above preparation processes are summarized and listed in Table 1.

## 4. Effect of Alloying Elements on Tissue Properties

After heat treatment, the mechanical properties and corrosion resistance of Cu–15Ni–8Sn alloys are greatly improved. In addition, a fourth element can be added to further improve the alloy’s properties. Several common alloying elements are listed in this section, and their effects on the microstructure and properties of the alloy are described. Intermetallic compounds with Ni will be formed when elements such as Si, Ti, and Nb are added to the alloy, which are diffusely distributed in the matrix, occupying the nucleation sites of discontinuous precipitation, inhibiting the movement of grain boundaries, and refining grains; thus, discontinuous precipitation is significantly inhibited. The precipitation of the DP phase is inhibited when elements such as Fe and Co are added, which solidly dissolve into the copper matrix as solute atoms and affect the organization and properties of the alloy by influencing the aging precipitation behavior.

### 4.1. Si

**Figure 6:**
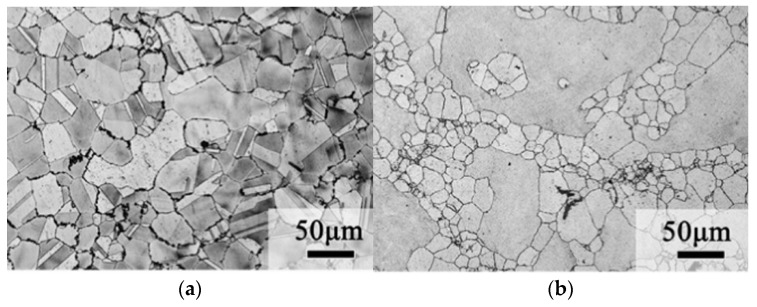
Structure of the extruded Cu–9Ni–2.5Sn–xSi alloy as shown by an optical microscope: (**a**) x = 0; (**b**) x= 0.5 [31].

The precipitation of phase γ′ in the matrix has been found to be delayed by the addition of Si, thus retarding the hardening of the matrix [30]. Fine particles precipitated from the Ni_31_Si_12_ phase were observed in the grain boundary and matrix of quenched samples with a Si content of more than 0.4%, and it is considered that these fine particles could occupy the nucleation position of crystal cells and inhibit the migration of grain boundaries so as to strengthen the matrix and fine grains. Luo [31] prepared low-Sn Cu–Ni–Sn alloys, which led to a reduced precipitation strengthening effect [32]. Therefore, Si and Al elements were added to the alloy and the results showed that the strength of the alloy could reach 861 MPa, comparable to that of Cu–15Ni–8Sn, and the alloy had an elongation of up to 18%. As shown in Figure 6, the average diameter of dynamic recrystallized grains of the 0 Si alloy is 46 ± 2.2 µm, and the average diameter of recrystallized grains of the 0.5 Si alloy is 34 ± 4.3 µm. This is due to the formation of Ni_3_Si nanoparticles by Si and Ni, which inhibits grain recrystallization and thus refines the grains. Moreover, due to the reduction of Sn, macroscopic segregation during the alloy preparation process is reduced or even eliminated, resulting in a more homogeneous microstructure.

### 4.2. Ti

On adding Ti, the Ni_3_Ti phase is formed during solidification [33], and the Ni_3_Ti phase does not dissolve in the subsequent solid solution treatment, leading to grain refinement due to the inhibitory effect of the Ni_3_Ti phase on grain boundary migration. Meanwhile, the nucleation sites of discontinuous precipitates are reduced on adding Ti, which inhibits the discontinuous precipitates.

**Figure 7:**
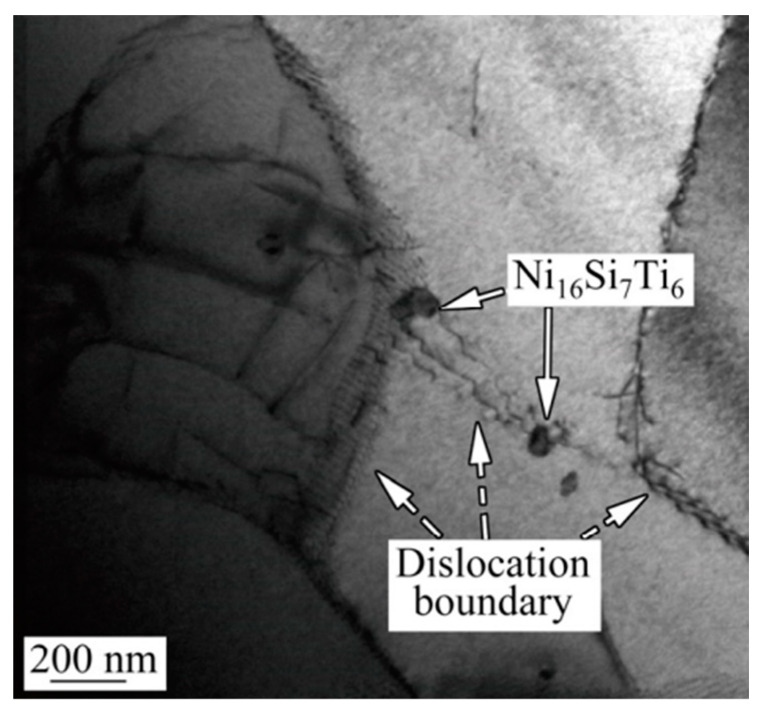
TEM image of Ni_16_Si_7_Ti_6_ particles and the dislocation boundary [35].

Generally, more than one element is added to improve the properties of materials. Zhao [34] added Ti and Si to a Cu–15Ni–8Sn alloy, which led to the precipitation of the Ni_16_Si_7_Ti_6_ intermetallic compound after hot extrusion and subsequent air cooling. Zhao found that (1) in the process of high-temperature hot extrusion, these kinds of micro- and nano-particles are evenly dispersed in the matrix and the nucleation of dynamic recrystallization is effectively promoted by the micron particles due to the particle-stimulated nucleation (PSN) effect, and (2) during the subsequent air cooling process, the growth of recrystallized grains is effectively inhibited by the nanoparticles through pinning grain boundary migration, accelerating the transformation from small to large angular grain boundaries [35]. Figure 7 is the TEM image of Ni_16_Si_7_Ti_6_ particles and the dislocation boundary. Thus, the addition of elements leads to fine grain strengthening and grain boundary strengthening. The tensile strength and elongation of the alloy can reach the values of 909 MPa and 30%, respectively, with significantly improved tensile properties.

### 4.3. Fe

In the late aging period, the properties of the alloy can significantly deteriorate due to the rapid nucleation and growth of discontinuous precipitates with a γ (DO_3_) structure. The growth of discontinuous precipitates was inhibited successfully by adding Fe [29]. According to Turnbull’s theory [36], the growth rate of DP (G) can be calculated by the following formula (Equation (1)):

(1) $$G=\frac{4\delta D_{b}}{S^{2}},$$

where δ is the cell boundary thickness and D_b_ and S are the solute diffusivity along the forward boundary and interlayer spacing, respectively. In this study, δ is unknown and is assumed to be constant.

**Figure 8:**
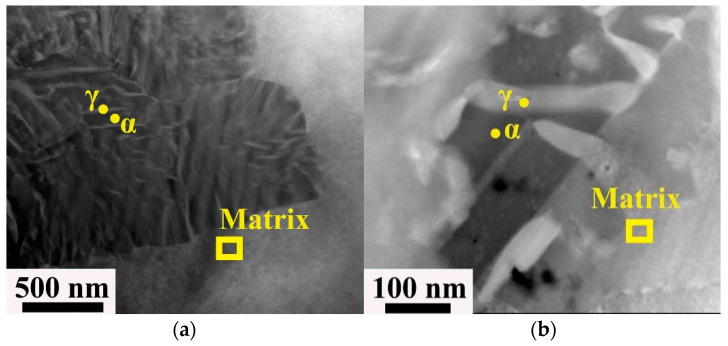
STEM image of a cold-rolled alloy Cu–15Ni–8Sn–xFe: (**a**) x = 0; (**b**) x = 0.5 [29].

The rapid diffusion of Fe atoms causes the preferential precipitation of Fe clusters in the discontinuous precipitates, which increases the interlayer spacing of DP. Therefore, after the addition of Fe, the D_b_ value decreases and the S value increases, which inhibits the further growth of discontinuous precipitates. As shown in Figure 8, the discontinuous precipitation of the γ phase was reduced significantly by the addition of Fe. This is because Fe is mainly enriched in the core of the DO_22_ ordered phase, which increases the stability of the DO_22_ ordered phase, making it difficult to transform the DO_22_ ordered phase to the γ (DO_3_) phase. Furthermore, the tensile strength of the material can reach a value of 1250 MPa, which is 123 MPa higher than that of the Fe-free alloy.

### 4.4. P

**Figure 9:**
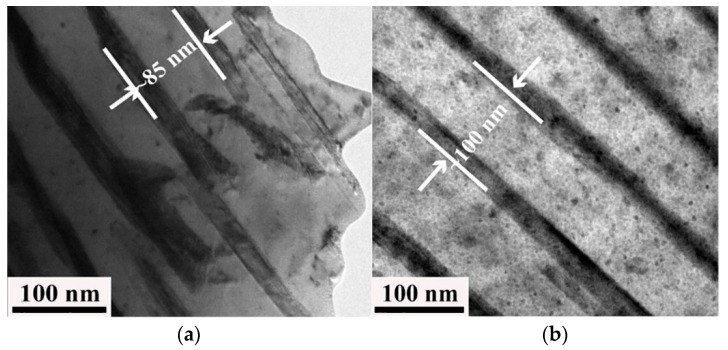
TEM bright-field image of the alloy Cu–15Ni–8Sn–xP: (**a**) x = 0; (**b**) x = 0.2 [37].

The addition of the trace element P (0.1 wt% and 0.2 wt%) can effectively inhibit the nucleation and growth of DP, but the effect on DP is reversed when the content of the element P is greater than 0.3 wt% [37]. This is because the nucleation position of DP is occupied by the Ni_10_SnP_3_ phase located at the grain boundary, which inhibits its nucleation. The grain can be refined by the addition of P. Although the content of the element P is larger, the grain boundary area fraction increases and the grain boundaries are the main location of DP nucleation. Thus, DP nucleation can be promoted by the increase in the grain boundary area fraction. In addition, Ni_10_SnP_3_ will precipitate at the grain boundary, absorbing solute atoms in the vicinity, and as a result of this, the surrounding matrix solute is impoverished and unable to precipitate ordered phases, which eventually results in the formation of a precipitation-free zone, which is also a reason for the inhibition of the DP phase by P. Figure 9 displays the TEM bright-field image of the Cu–15Ni–8Sn–xP alloy. The interlayer spacing of the DP phase is indicated by the scale in the figure, and it is found that the interlayer spacing of the DP phase increases after the element P is added. As seen from Equation (1), the interlayer spacing increases and the DP growth rate decreases.

### 4.5. V

**Figure 10:**
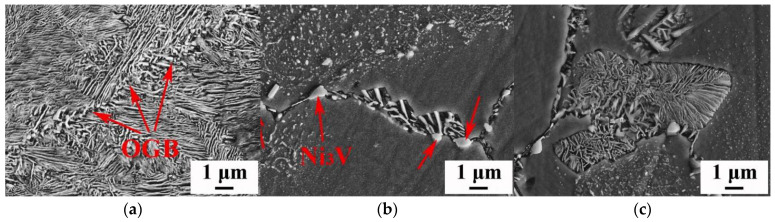
SEM image of discontinuous precipitation at grain boundary of Cu-15Ni-8Sn-xV alloy (**a**) x = 0; (**b**) x = 0.4; (**c**) x = 1.0 [38].

The grains can be refined by adding the element V to Cu–15Ni–8Sn. When the V content is less than or equal to 0.4 wt%, grain refinement is mainly limited by growth. When the V content increases to 0.6 wt%, the grain refinement mechanism changes to heterogeneous nucleation [38]. The production of DP can also be inhibited by the addition of V [39]. The original grain boundary (OGB) and layered structure can be clearly seen in Figure 10. When no V is added, discontinuous precipitation occurs preferentially at the grain boundary and grows smoothly toward the matrix. The precipitated Ni_3_V particles are located at the grain boundary, as shown by the red arrows in the figure. These particles occupy the nucleation position of discontinuous precipitation. In addition, the Ni_3_V particles precipitated at the grain boundary have an obvious pinning effect on the grain boundary, increasing the activation energy of DP and making DP precipitation difficult. The addition of V has a significant effect on the interlayer spacing of DP in the Cu–15Ni–8Sn alloy, and the interlayer spacing of DP increases with the increase in the V content. When the V content is 0.4 wt%, the alloy’s resistance to softening can be improved with appropriate heat treatment and the tensile strength can reach a value of 990 MPa [38]. The OGB and layered structure can be clearly seen in the figure. When V is not added, discontinuous precipitation occurs preferentially at the grain boundary and grows smoothly toward the matrix.

### 4.6. Co

**Figure 11:**
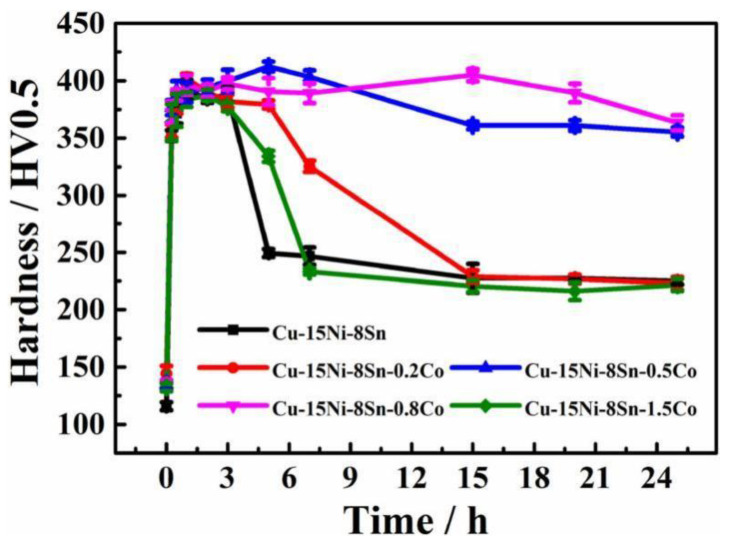
Aging hardening curves of alloys with different Co contents treated by 773 K isothermal aging [40].

Co addition can inhibit DP production, but the mechanism is different from those above. On adding Co, the dendritic structure of the alloy becomes shorter, the bony Sn-rich region becomes smaller, the Sn content in the matrix increases, and the as-cast structure of the alloy is more uniform. At this point, the alloy is harder than the alloy without the addition of Co. This is because Co more readily binds to vacancies. The lack of vacancies of Sn and Ni atoms in Cu is caused by the addition of Co, and the segregation of Sn clusters is suppressed [40], increasing the activation energy of the alloy and delaying the formation of DP. In addition, Co atoms are dissolved in the core of the L1_2_ phase, which enhances the stability of the L12 phase and thus inhibits the conversion of L1_2_ to DO_3_ at the later stage of heat treatment. The curve of microhardness (HV) of the Cu–15Ni–8Sn–xCo alloy with an aging temperature of 773 K is shown in Figure 11. Adding the proper amount of Co to the alloy can improve the hardness, delay the aging hardening behavior, and improve the aging resistance of the alloy.

### 4.7. Nb

The strength and toughness of the alloy Cu–15Ni–8Sn can be improved by the addition of Nb. In a study carried out by Ouyang et al. [25], some intergranular precipitates in the Cu–15Ni–8Sn alloy rich in Ni, Sn, and Nb were observed through high-angle annular dark-field imaging analysis. Ouyang et al. suggested that these precipitates contribute to the alloy’s strength and toughness. Chen et al. [41] concluded that the increase in strength and toughness of the Cu–15Ni–8Sn alloy is due to the formation of NbNi_2_Sn and NbNi_3_ phases, that the increase in strength is mainly due to grain boundary and precipitation strengthening, and that the increase in toughness is mainly due to grain refinement. One of the studies carried out by Gao [42] found that the addition of Nb could refine the grain, forming the NbNi_3_ compound and improving the strength and toughness of Cu–9Ni–6Sn alloys.

Effect of alloy elements on microstructure and properties are summarized in Table 2.

## 5. Effect of Plastic Deformation and Heat Treatment on the Microstructure and Properties of Cu–Ni–Sn Alloys

Cu–15Ni–8Sn alloys have good mechanical properties and corrosion resistance. These alloys are often prepared by casting or other processes involving plastic deformation or heat treatment which can bring out more excellent properties of the alloys. The most important parameter of plastic deformation is the amount of deformation. If the deformation is too low, it will not achieve the effect of strengthening, and if the deformation is too high, it will lead to the precipitation of the DP phase, leading to a decline in the mechanical properties and corrosion resistance. Therefore, the entire treatment process should be controlled by controlling the deformation according to the alloy and with heat treatment. In the process of heat treatment, tempering temperature and time are particularly important. If the temperature or time is too low to achieve the effect of aging, the alloy will not be strengthened. However, if the temperature is too high, it will lead to over-aging and the mechanical properties of the alloy may be reduced.

### 5.1. Plastic Deformation

Deformation treatment is an important method of strengthening alloys. For Cu–Ni–Sn alloys, the higher the Sn content, the higher the pre-cold processing level should be. In addition, the momentum of continuous transformation is increased by cold working, which is conducive to the subsequent transformation of the reinforced phase precipitation [43]. Deformation also affects the microstructure of the alloy. The deformation after homogenization influences the microstructural changes during aging. The dislocation created during deformation and the spinodal structure interact, accelerating the process of phase transformation of the aging alloys. The deformation before aging promotes the aging microstructure changes and rapidly increases the size of forming phase [44].

**Figure 12:**
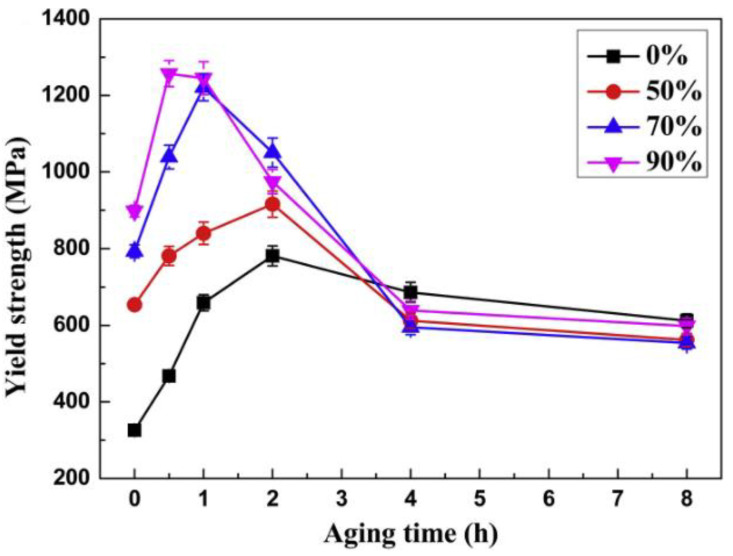
The variations in yield strength versus aging time after aging treatment at 400 °C [45].

One of the studies carried out by Guo [45] investigated the effect of deformation on the microstructure and properties of the alloy during cold rolling. After pre-rolling, the surface oxide scale is removed by heating the alloy to 850 °C. Then, the alloy is rolled at room temperature with different deformations, and finally tempered at 400 °C. With the increase in deformation, the tempering peak time is greatly reduced, reducing the cost and significantly improving performance. Especially at 90% deformation, the yield strength of the alloy can reach a value of 1256 MPa after aging, and the main strengthening mechanisms are solution strengthening, dislocation strengthening, and precipitation strengthening. Figure 12 shows the yield strength of the alloy with different rolling deformations. It is worth noting that when the alloy deformation increases, DP is also more likely to precipitate, and the intracrystalline precipitation of DP is transferred to the grain boundaries, which may make the grain boundary more susceptible to corrosion. Hot extrusion is a method of heat treatment, and the temperature of hot extrusion is slightly lower than the quenching temperature. For this alloy, the hot extrusion temperature is about 830 °C. In the process of hot extrusion, due to the high temperature dynamic strain, aging (DSA) occurs [46]. DSA is the result of dynamic interactions between mobile dislocations and diffusible solutes. For Cu–Ni–Sn alloys, the element Sn is the diffusible solute in DSA, and DSA causes Sn and Ni to polarize. In hot extrusion (which is the strengthening phase L1_2_), Ni3Sn is precipitated during the subsequent aging. Due to the dislocation pinning effect produced by DSA, the spinodal decomposition in subsequent aging is inhibited, but the time of aging is considerably reduced. It is also noteworthy that hot extrusion also promotes DP precipitation. Therefore, the strengthening effect of hot extrusion on alloys remains to be fully investigated. In addition, it has been shown that the grain size exhibits heritability, i.e., on the basis of the preparation of fine grains, the mechanical properties of the alloy can be further improved by the subsequent operation of plastic deformation, mainly due to additional dislocations added by the grain refinement [47].

Good corrosion resistance is another property of Cu–15Ni–8Sn, and it is also of great significance to study the effect of heat treatment on this property. A study carried out by Zhang [48] investigated the effect of different thermomechanical treatments on the seawater corrosion resistance of the alloy and found that cold rolling after hot rolling reduces the precipitation of the discontinuous Sn- and Ni-rich phases and avoids the occurrence of local corrosion. This multi-stage thermomechanical treatment increases the amount of deformation in the material, resulting in more nano-sized ordered precipitates, which are more uniformly distributed in the subsequent aging process and provides better corrosion resistance. A heat treatment process for the alloy involving cold rolling and hot rolling was also carried out by Yang [49]. The difference between the studies by Yang [49] and Zhang [48] is that Yang performed two-stage homogenization annealing prior to hot rolling (800 °C × 4 h + 830 °C × 4 h) and the differences between peak aging and over-aging alloy properties were investigated. The annual average corrosion rate after peak aging treatment is 0.01529 mm/a, which is lower than that of over-aged samples. This is because over-aging causes more DP phases to precipitate at the grain boundary, forming a micro-galvanic cell with the matrix and accelerating the corrosion of the alloys.

### 5.2. Tempering

**Figure 13:**
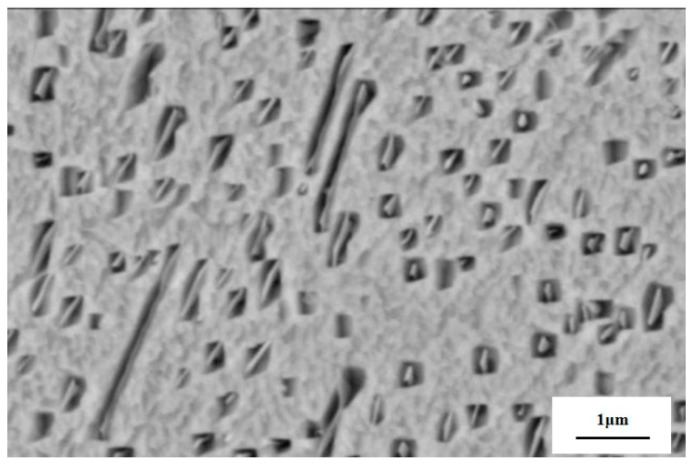
SEM images of the precipitated strengthening phase from γ grains in the aged Cu–Ni–Sn alloy [51].

After an alloy has been prepared by processes such as casting and powder metallurgy, it is often subjected to quenching and tempering, during which the strengthening phase is precipitated and the grains are refined. Generally, the alloy is first heated to 850~830 °C in a vacuum, followed by 4 h of water quenching. The choice of tempering temperature is particularly important. A study carried out by Hu [50] explored the effects of tempering temperature and tempering time on the microstructure and properties of the alloy. After the same tempering time, the hardness of the alloy was the highest at a tempering temperature of 350 °C, reaching 32 HRC with a strength of up to 900 MPa. The tempering time was explored at this temperature, and the hardness peaked at an aging time of 1.5 h and then decreased further with time. The increase in hardness is due to the decomposition of the solid solution into fine amplitude-modulated zones, and the decrease in hardness at a later stage is due to the creation of the soft new phase γ (DO_3_). Figure 13 is the SEM image of the precipitated strengthening phase from γ grains. This was also confirmed in the study by Cong [51], who found that the higher the solution temperature, the earlier the peak of age strengthening. Cong also conducted heat treatment research on the Cu–15Ni–8Sn alloy and found that after solid solution and aging treatment (800 °C + 400 °C × 4 h), the test alloy can achieve a hardness of 35 HRC and a tensile strength of 1300 MPa.

### 5.3. Heat Treatment Process of Cu–15Ni–8Sn Prepared by LPBF

**Figure 14:**
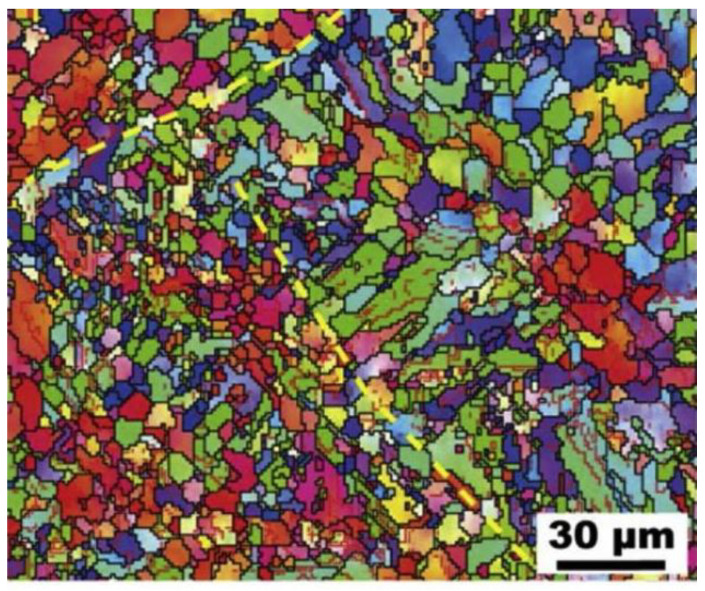
Electron backscatter diffraction image of LPBF [55].

LPBF is a new preparation process. As shown in Figure 14, the microstructure has obvious differences from that of ordinary casting, namely that the microstructure is composed of fine equiaxed crystals and columnar crystals. Thus, the heat treatment process is different from that of an ordinary casting alloy. Moreover, the preparation of Cu–15Ni–8Sn alloys by LPBF is still in the research stage, and there has been less research into the heat treatment of the alloys. Due to the rapid cooling rate of the LPBF process and the low precipitation of the alloy, a heat treatment process is required to strengthen the alloy. At present, the heat treatment process is divided into solid solution aging and direct aging. The DO_22_ and L1_2_ ordered phases are further precipitated after aging. It is found that after direct aging, the alloy has a higher yield strength, which is due to the retention of the initial Sn-rich precipitation phase produced by the LPBF process when precipitation strengthening dominates, while solid solution aging causes better plasticity, which is due to the annealing twinning to inhibit the precipitation of DP during aging. The aging time is generally 0.5 to 1 h, and it should not be too long. Otherwise, the layered DP begins to nucleate and grow slowly, resulting in intergranular fracture [52]. If plasticity is not considered, direct aging treatment is an economic and environmentally friendly method for preparing high-strength LPBF-treated Cu–15Ni–8Sn alloys [53], which is potentially instructive for improving the mechanical properties of other LPBF-treated precipitated phase-strengthened alloys.

As per Wang [54], the LPBF process shows promise in the manufacture of easily segregated Cu–15Ni–8Sn alloys. The solubility limit of the solute in the Cu–15Ni–8Sn alloy is substantially elevated. In addition, numerous slender cellular structures were observed in the LPBF-manufactured alloy, and many dislocations and dislocation tangles appeared around the precipitate dispersed between cellular structures. Thus, the LPBF-manufactured Cu–15Ni–8Sn alloy exhibited a good combination of strength and toughness.

## 6. Applications of Cu–Ni–Sn System Alloys

As a new generation of elastic materials, Cu–Ni–Sn system alloys are widely used in the electronic communication industry, aerospace, integrated circuits, and the instrumentation industry. Where traditional copper-based elastic alloys have failed to meet the various performance requirements, Cu–Ni–Sn alloys meet the higher performance requirements for copper-based elastic materials: (1) these alloys are strong, and the size of the components can be reduced to save space; (2) in terms of the long working hours of the equipment, components made from these alloys have excellent contact and reliable stability; and (3) in different working environments, the components have high temperature resistance, corrosion resistance, and other properties. Meanwhile, due to the low production cost, non-toxicity, high thermal stability, and high temperature strength, these alloys are considered as one of the substitutes for beryllium copper, with great potential in the field of non-ferrous elastic alloys [56].

In the 1970s, both domestic research and research abroad was carried out on Cu–Ni–Sn system alloys and a series of advances were made [43,57]. In the 1980s, a variety of Cu–Ni–Sn alloys were incorporated into production technology standards in the US, which greatly promoted the commercial application of the alloys. Domestic research on Cu–Ni–Sn alloys also started earlier, and the gap between domestic research and that performed internationally on alloy organization, mechanics, and electrical properties is not large. There has been more research on the application of the alloys, which has promoted the development of beryllium copper substitute materials.

Zhao [58] prepared a Cu–15Ni–8Sn alloy using the vacuum melting method. The mechanical and physical properties of the resulting material are comparable to those of beryllium copper, and the conductive contact reeds made by punching successfully passed both electrical life and mechanical life tests and were introduced into industrial production in bulk for use in the LXW5 series of microswitches. The following year, Zhao [59] prepared a Cu–9Ni–6Sn alloy using the same method. The alloy was applied to conductive contact reeds, and its performance also met the requirements. Moreover, an age-hardened high-strength, high-elasticity, and corrosion-resistant deformed copper alloy was successfully prepared by Zhang [60] by multi-component alloying of copper–nickel–tin alloys, and it is being used as a novel elastic alloy material. This Cu–Ni–Sn alloy is suitable for making plates, strips, rods, wires, etc., for use in radio, instrumentation, electrical, and other industries as electrically conductive pop-ups and contact reeds. This type of Cu–Ni–Sn alloy is widely used and has obvious advantages: (1) The alloy has a high fatigue life. In one study, it was used as a strip conductive spring and loaded into the actual instrument to test the fatigue life. At the end of the experiment, the reed was not damaged nor fractured and still continued to work. In addition, the spring fatigue life was several times higher than the design requirements. (2) The alloy has a low stress relaxation rate. Compared with beryllium copper reeds, the stress relaxation rate of both reeds is 3.5%, which meets design requirements. (3) The alloy has a high wear life. In addition, the corrosion resistance of this alloy was found to be slightly better than that of C72700 under the same experimental conditions. Thus, the above results show that the new Cu–Ni–Sn alloy can partially replace the original beryllium bronze and its precious metal composites.

Not only do Cu–Ni–Sn alloys have high elasticity and electrical conductivity, but they also have high strength and wear and corrosion resistance. These alloys are a key basic material for core components in the aerospace industry, heavy-duty equipment, petrochemicals, high-grade CNC machine tools, and robotics [61]. Compared to conventional moderate-strength aluminum bronze, Cu–Ni–Sn is considered to be an improved bearing alloy as it combines high strength and good tribological characteristics [62]. As a modern bearing material, the Cu–Ni–Sn alloy has become the material of choice for bushings or plain bearing components. In particular, Cu–15Ni–8Sn, commercially known as ToughMet 3, is an alternative or upgrade to conventional medium- to high-strength aluminum bronze bearing alloys. Krus [63] carried out comparative evaluation tests of ToughMet 3 and leaded tin bronze for bearing materials as well as gasket materials. In the wear experiments, ToughMet 3 CX105 showed the lowest wear rate of 0.1 mg/h under normal lubrication conditions, while C93200 showed the highest wear rate of 1.6 mg/h, with the surface of the tin bronze alloy significantly darkened at the end of the experiment. Under limited lubrication conditions, the bronze specimens showed severe distortion at the edges.

The mechanical properties of ToughMet 3 are significantly better than those of leaded tin bronze, as shown in Table 3 [63].

The high yield strength and oxidation resistance of ToughMet make it a candidate for heavy equipment transmission and drive applications. Compared to leaded tin bronze C93200 and C93700, ToughMet has similar frictional properties but is superior in resisting surface deformation, rises in temperature, lubricant degradation, and oxide growth.

The Cu–Ni–Sn alloy can be used as a corrosion-resistant steel due to its excellent corrosion resistance, and it greatly improves the corrosion resistance of hull steel in a strongly acidic chloride environment. Cu is an effective element for improving the corrosion resistance of steel. Its main mechanism is enrichment on the surface of steel in the form of redeposited particles, which passivate the steel matrix, reduce the solubility of steel, and inhibit the nucleation of pitting corrosion. The corrosion resistance of the steel will be further enhanced by Sn. The main mechanism of action is the formation of a dense protective film of corrosion products on the surface of the steel, which effectively prevents any interaction between the substrate and the corrosive medium and inhibits the corrosion of the steel in the medium [64].

## 7. Summary

In this section, a preliminary summary of the Cu–Ni–Sn alloy is presented in terms of preparation processes, strengthening mechanisms, etc. The conclusions are as follows:

(1) As a copper-based highly elastic conductive alloy, it can be used in precision instrumentation and the electrical industry to prepare various elastic components, such as electronic connectors, springs, bearings, and bushings. In terms of preparation processes, although element segregation is caused by continuous casting, a large amount of segregation can be eliminated through continuous casting modifications, for example, by the incorporation of electromagnetic stirring technology or by subsequent heat treatment. At present, continuous casting is still an economical and effective preparation process. In addition, powder metallurgy is a good preparation process and can be applied in production. LPBF technology is still in the experimental stage currently. However, whereas mechanistic studies are less transparent and difficult to apply in practice due to excessive costs and the absence of systematic process parameters, LPBF technology has shown its potential;
(2) The factors affecting the properties of Cu–15Ni–8Sn alloys are grain size, distribution, and the size of the second phase, in addition to the main factor, i.e., the precipitation of the DP phase. This is a brittle phase, and the mechanical properties and corrosion resistance of the alloy will be negatively affected, especially at the grain boundary. Grains can be refined, and the precipitation of the DP phase can be suppressed by adding alloying elements such as V, Ti, and Co. Various elements have different mechanisms of action, and some elements can also promote the precipitation of the fine dispersed second phase, further enhancing the mechanical properties of the alloy. However, there are obvious deficiencies in the research on microalloying of B, Zr, Mn, and other elements, and there is a lack of systematic analysis and detailed discussion, which is worthy of further discussion in follow-up research. The purpose of heat treatment is to minimize the precipitation of the discontinuous DP phase, but some heat treatment methods have the opposite effect. Therefore, to improve the overall performance of the alloy, it is necessary to match the alloy with its application environment;
(3) It can be seen from this review that Cu–Ni–Sn alloys have good mechanical properties, with a tensile strength of up to 1300 MPa. Thus, they can be called ultra-high-strength alloys. Therefore, these alloys have a wide range of applications, which will bring more challenges for the alloys to overcome. For example, the requirements of coastal areas or marine engineering regarding the corrosion resistance of alloys are strict and the economic loss caused by corrosion is also large. However, there is little research on the corrosion resistance of the alloys, especially those prepared by LPBF technology. Therefore, a concerted effort is required to fill this gap in the studies of corrosion resistance mechanisms of alloys.

## Figures and Tables

**Table 1:** The advantages and disadvantages of various methods of preparing Cu–Ni–Sn alloys.

Preparation Process	Advantages	Disadvantages
Continuous casting	Economical and efficient	Presence of elemental segregation
Powder metallurgy	Homogeneous composition and fine grain size	High cost but only suitable for simple shapes
Plasma spraying	Finer grain, higher density, and near-final formation	High cost and a wide variation in different microstructures
Rapid solidification	Refined grain, suppressed segregation, high material utilization, and simple process	High cost, complex parameter control, and large residual stresses
Vacuum melting	Guaranteed high purity of materials	High equipment requirements and difficult to apply in industry

**Table 2:** Effect of alloy elements on microstructure and properties.

Element	Strengthening Phase	Strengthening Mechanism	Mechanical Properties
Si	Ni_31_Si_12_, Ni_3_Si	Strengthening of the grain boundary and inhibition of grain recrystallization	Tensile strength: 861 MPa
Ti	Ni_3_Ti, Ni_16_Si_7_Ti_6_	Strengthening of the grain boundary, inhibition of DP phase precipitation, and strengthening of fine grains	Tensile strength: 909 MPa
Fe	--	Inhibition of DP phase precipitation and increase in the stability of the DO_22_ ordered phase	Tensile strength: 1282 MPa
P	Ni_10_SnP_3_	Tensile strength: 1282 MPa	Hardness: 400 HV
V	Ni_3_V	Refinement of grains and inhibition of DP phase precipitation	Tensile strength: 990 MPa
Co	--	Inhibition of DP phase precipitation and enhancement of L1_2_-phase stability	Hardness: 412 HV
Nb	NbNi_2_Sn, NbNi_3_	Strengthening of the grain boundary and strengthening of precipitation	Tensile strength: 590 MPa

**Table 3:** Properties of different alloys.

Materials	Yield Strength (MPa)	UTS (MPa)	Elongation (%)	Hardness (HB)
ToughMet 3 CX105	724	758	4	294
C93200	124	241	20	60
C93700	124	241	20	60

## Data Availability

Not applicable.
